# Differentiation of acanthamoeba keratitis from other non-acanthamoeba keratitis: Risk factors and clinical features

**DOI:** 10.1371/journal.pone.0299492

**Published:** 2024-03-12

**Authors:** Shaker Osaywid Alreshidi, José Manuel Vargas, Khabir Ahmad, Ahmed Yousef Alothman, Eman D. Albalawi, Abdulmohsen Almulhim, Saad Hamdan Alenezi, Hani Basher ALBalawi, Naif Mamdouh Alali, Faris Hashem, Mohanna Aljindan

**Affiliations:** 1 Ophthalmology Department, College of Medicine, Majmaah University, Majmaah, Saudi Arabia; 2 Cornea, External Diseases Section, University of Texas Southwestern Medical Center, Dallas, Texas, United States of America; 3 Ophthalmology Division, King Abdullah Bin Abdulaziz University Hospital, Riyadh, Saudi Arabia; 4 Research Department, King Khaled Eye Specialist Hospital, Riyadh, Saudi Arabia; 5 Ophthalmology Department, Imam Abdulrahman Bin Faisal University, Dammam, Saudi Arabia; 6 Clinical Sciences Department, College of Medicine, Princess Noura Bint Abdulrahman University, Riyadh, Saudi Arabia; 7 Department of Ophthalmology, College of Medicine, Jouf University, Sakakah, Saudi Arabia; 8 Department of Surgery, Division of Ophthalmology, Faculty of Medicine, University of Tabuk, Tabuk, Saudi Arabia; University at Buffalo Jacobs School of Medicine and Biomedical Sciences: University at Buffalo School of Medicine and Biomedical Sciences, UNITED STATES

## Abstract

**Introduction:**

Infectious Keratitis is one of the most common ocular emergencies seen by ophthalmologists. Our aim is to identify the risk factors and clinical features of Acanthamoeba Keratitis (AK).

**Methods:**

This retrospective chart review study was conducted at King Khaled Eye Specialist Hospital, Riyadh, Saudi Arabia, and included all the microbial keratitis cases, male and female patients of all ages. The main outcome is the differentiation between various microbial keratitis types.

**Results:**

We included 134 consecutive eyes of 126 persons. We had 24 cases of acanthamoeba keratitis, 22 bacterial keratitis, 24 fungal keratitis, 32 herpetic keratitis, and 32 bacterial co-infection. Contact lens wear was found in 33 eyes (24.6%). Among acanthamoeba keratitis patients, 73% were ≤ 39 years of age, and 73% were females (P <0.001). Also, in AK cases, epithelial defect was found in all cases (100%), endothelial plaques were found in 18 eyes (69.2%), 12 cases had radial keratoneuritis (46.2%), and ring infiltrate was found in 53.8% of AK cases.

**Conclusions:**

We determined the factors that increase the risk of acanthamoeba infection and the clinical characteristics that help distinguish it from other types of microbial keratitis. Our findings suggest that younger females and patients who wear contact lenses are more likely to develop acanthamoeba keratitis. The occurrence of epitheliopathy, ring infiltrate, radial keratoneuritis, and endothelial plaques indicate the possibility of acanthamoeba infection. Promoting education on wearing contact lenses is essential to reduce the risk of acanthamoeba infection, as it is the most significant risk factor for this infection.

## Introduction

Infectious keratitis is one of the most common ocular emergencies presented to the ophthalmology clinic. Several etiologies have been identified, and microorganisms are the most common causes. Distinguishing the causative organism is essential in proceeding with the proper antimicrobial therapy to ensure the best outcome. Acanthamoeba infection of the cornea has a wide spectrum of corneal manifestations requiring different management strategies. It can have devastating consequences if not managed early. There are various challenges in management, including misdiagnosis, inappropriate antimicrobial treatment, use of topical steroids prior to correct diagnosis, and the presence of other secondary organisms. Recently, acanthamoeba has been reported in about 5% of contact-lens-associated keratitis, and the prevalence increased with the use of contact lenses [1].

Acanthamoebas are good opportunists due to their ability to encyst, which results in being more resistant to antimicrobial agents. Without appropriate early treatment, acanthamoeba progresses deeper into the corneal stroma and widely to the sclera. Acanthamoeba is presumed to feed on keratocytes, especially in the deep stroma, leading to corneal destruction, increased resistance because of cyst formation, and less drug penetration. Severe cases lead to corneal melting, scleral involvement, glaucoma, and cataracts, which are more common in late-presenting stages or poorly managed cases. A higher proportion of severe cases end with poor visual acuity [2].

The laboratory investigation, including staining and culturing techniques required for identifying acanthamoeba, needs an expert microbiologist. Confirmation of the diagnosis is particularly important to distinguish acanthamoeba from bacterial, herpetic, and fungal keratitis since the treatment is different. Corneal scraping for staining and culture is considered the gold standard for identifying the causative pathogen [3, 4]. Staining may reveal false negative results, which require biopsy for identification of Acanthamoeba tested through smears and PCR [5, 6]. One very useful tool for the identification of Acanthamoeba is In Vivo Confocal Microscopy (IVCM) [7]. IVCM is a non-invasive imaging modality and is the most sensitive technique for acanthamoeba keratitis diagnosis over PCR and culture [8]. The sensitivity and specificity of IVCM in identifying Acanthamoeba were up to 85.3% and 100%, respectively [9].

In our study, we focused on identifying Acanthamoeba risk factors and clinical features that may aid in the early differentiation of Acanthamoeba keratitis from other causes of keratitis.

## Materials and methods

The study was approved by the institutional review board (IRB) committee at King Khaled Eye Specialist Hospital, and a waiver for participation consent was obtained (IRB approval number: 2089-R). The study was performed in accordance with the Helsinki Declaration of 1964 and its later amendments.

This is a study of diagnostic accuracy. Retrospective electronic chart review of all the confirmed microbial keratitis cases, male and female patients of all ages, seen at King Khaled Eye Specialist Hospital (KKESH) in Riyadh, Saudi Arabia, between January 2014 and December 2020.

De-identified data were accessed by the authors on the 13^th^ of July 2020. A structured data sheet was used for data collection. Data were collected from medical records by the research primary investigator (PI) with the following variables: Date of birth, sex, eye involved, history of wearing contact lenses, comorbidities, presenting signs, the time between the onset of symptoms and presentation, visual acuity at presentation, previous treatment if any, laboratory findings, ancillary test results, imaging, and visual outcome. We excluded the cases with a lack of chart details or investigation documentation.

Data were entered in Microsoft Access and analyzed using Stata version 16. College Station, TX: StataCorp LLC. Mean, median, standard deviation and interquartile range were computed to describe continuous data as appropriate. Frequencies and percentages were computed to describe categorical data. Normally distributed data were compared using Student’s t-test, and non-normally distributed data were compared using the Mann-Whitney U test. Proportions were compared using chi-squared or Fisher’s exact test. A p-value less than 0.05 was considered statistically significant.

The complete dataset used in this study is available in the Supplementary information (S1 Dataset).

## Results

A total of 134 consecutive eyes of 126 persons were included. We identified 24 cases of acanthamoeba keratitis, 22 cases of bacterial keratitis, 24 fungal keratitis, 32 herpetic keratitis, and 32 bacterial co-infection (which includes 19 mixed fungal and bacterial keratitis, 11 herpetic mixed with bacteria, and two acanthamoebae mixed with bacterial keratitis) from the same period of time. The follow-up period ranged from 1 to 120 months, with a mean (SD) of 26.30 ± 19.84 months. There were 84 (62.7%) males and 50 (37.3%) females. Cases with age ≥ 40 were (59.7%). Left eyes were 78 (58.2%), and bilateral eyes were in 4 patients (3.17%). Fifty-three cases (39.5%) were associated with systemic diseases, mostly DM (38, 71.6%), 45 eyes had a history of ocular disease (40.2%), a history of herpetic disease was found in 29 eyes (21.6%), and a history of ocular trauma induced infection was found in 14 eyes (10.4%). Previous ocular surgery was in 39 eyes (29.1%), Contact lens wear was found in 33 eyes (24.6%), and 67 eyes (50%) were referred to as microbial keratitis. Also, ten eyes (7.5%) were reported using topical steroids along with topical antimicrobial medication.

Out of the acanthamoeba cases (n = 26), 19 eyes (73%) were ≤ 39 years of age (P <0.001), females were 19 (73%) (P <0.001), 11 right eyes and 15 left eyes. None were associated with Diabetes mellitus, P<0.001, and only two were associated with other systemic diseases (i.e. hypertension). Three eyes were associated with a previous history of herpetic eye disease (11.5%), none had a history of ocular trauma, 14 eyes were referred to our emergency department (53.8%), and 25 eyes were of contact lens (CL) wearers (96.1%) (P <0.001).

Overall, contact lens wearers, based on gender, were 13 males and 20 females. Soft contact lenses were used on 32 eyes (97.0%), daily soft contact lenses were used on 25 eyes (75.8%), and practicing proper CL hygiene was only in one case. We found that the majority of acanthamoeba keratitis cases were using contact lenses (95.83%) (P<0.001). In addition, among the CL wearers, 23 eyes had acanthamoeba keratitis (69.7%) (P<0.001). Contact lens use was reported in 100% of bilateral AK cases.

Among all the acanthamoeba keratitis (AK) cases, the median time from symptom onset to the initial visit was nine days ±11.9 (range: 2–60 days), which is longer than non-acanthamoeba keratitis (non-AK) cases, seven days ±16.5 (range: 2–120) with no statistically significant difference. There was no statistically significant difference in the interval of the start of care seeking (beginning of symptoms) and diagnosis between AK and non-AK cases; 57.7% of AK and 67.5% of non-AK were diagnosed in less than two weeks.

Our study found no statistically significant difference between acanthamoeba and non-acanthamoeba keratitis cases in the visual acuity (VA) at presentation. Twenty eyes of acanthamoeba keratitis, bacterial, and herpetic infections were presented and discharged with a VA of 20/100-20/200. While the higher frequency of fungal infection (7 eyes) presented and discharged with a VA of light perception (LP).

**Fig 1:**
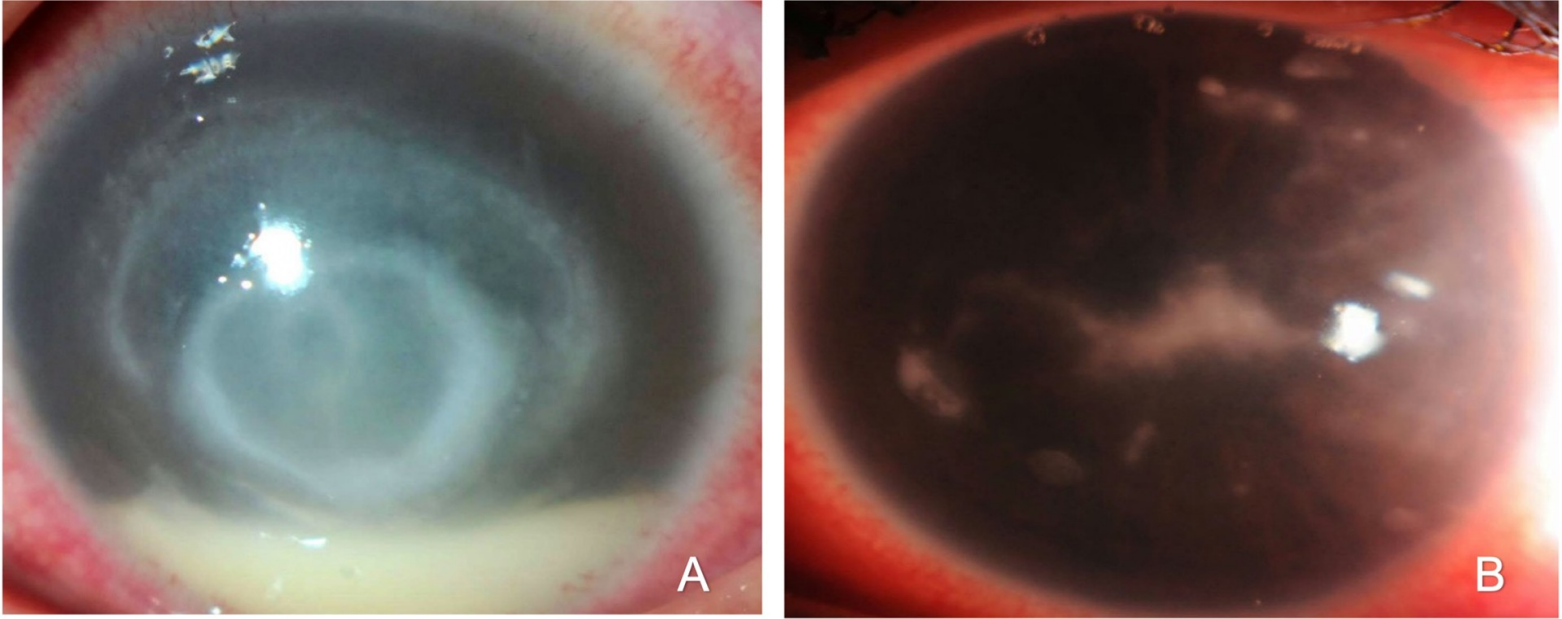
Slit-lamp photograph showing ring infiltrate lesion associated with acanthamoeba keratitis (A); Slit-lamp photograph showing retro-corneal plaques (endothelial plaques) lesion associated with acanthamoeba keratitis (B).

**Fig 2:**
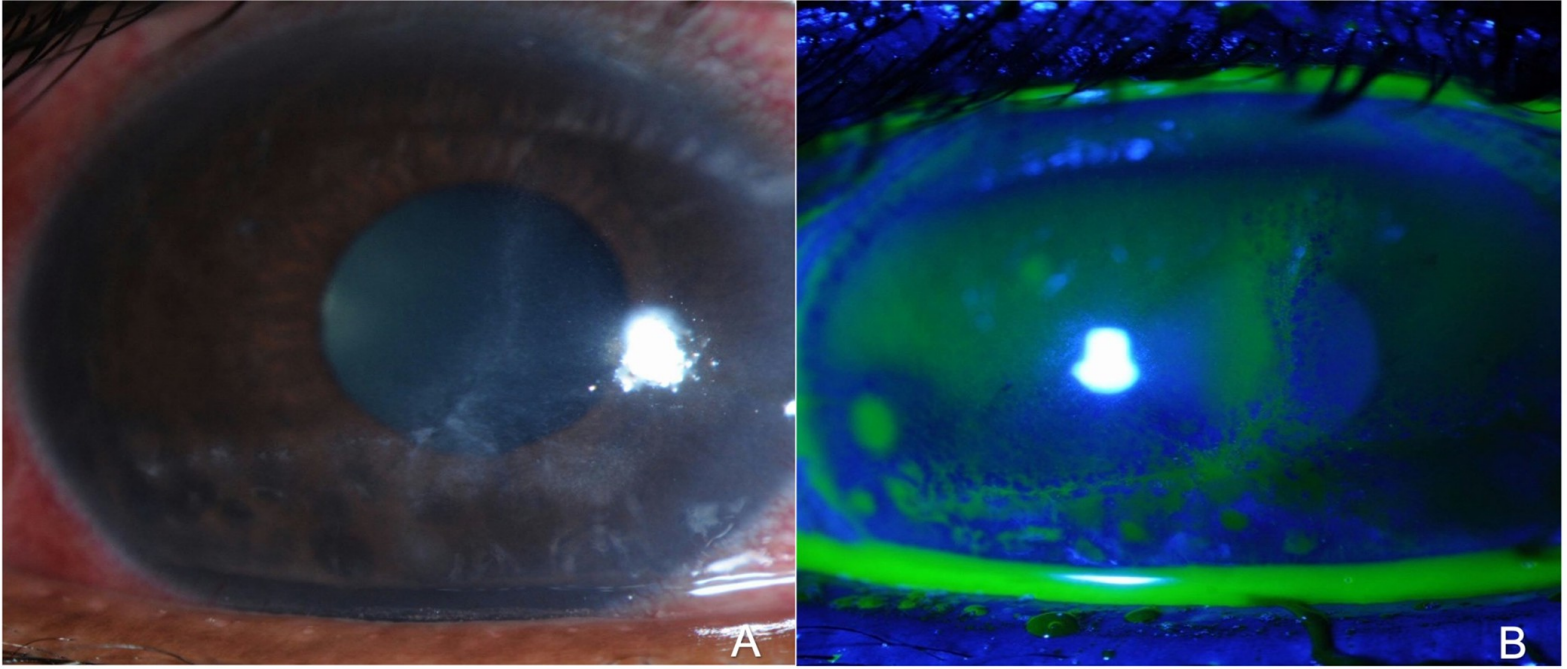
Slit-lamp photographs showing epitheliopathy lesion associated with acanthamoeba keratitis (A); fluorescein stain highlighting the epitheliopathy lesion (B).

The epithelial defect was found in 121 cases (90.2%), endothelial plaques in 23 (17.1%), radial keratoneuritis in 13 (9.7%), epitheliopathy in 6 (4.4%), ring infiltrate in 15 (11.2%). (Figs 1 and 2)

In Acanthamoeba keratitis cases, an epithelial defect was found in all acanthamoeba cases (100%), endothelial plaques were found in 18 eyes (69.2%), and 12 cases had radial keratoneuritis (46.2%). In comparison, cases with both endothelial plaques (EP) and perineuritis (PN) were all acanthamoeba keratitis cases. Out of all infectious keratitis cases, ring infiltrate was found in 15 eyes; 14 of them were acanthamoeba keratitis (93.3%), and one eye had herpetic confirmed with PCR. Epitheliopathy was found in 6 cases of all samples, and all cases were acanthamoeba keratitis (100%).

**Fig 3:**
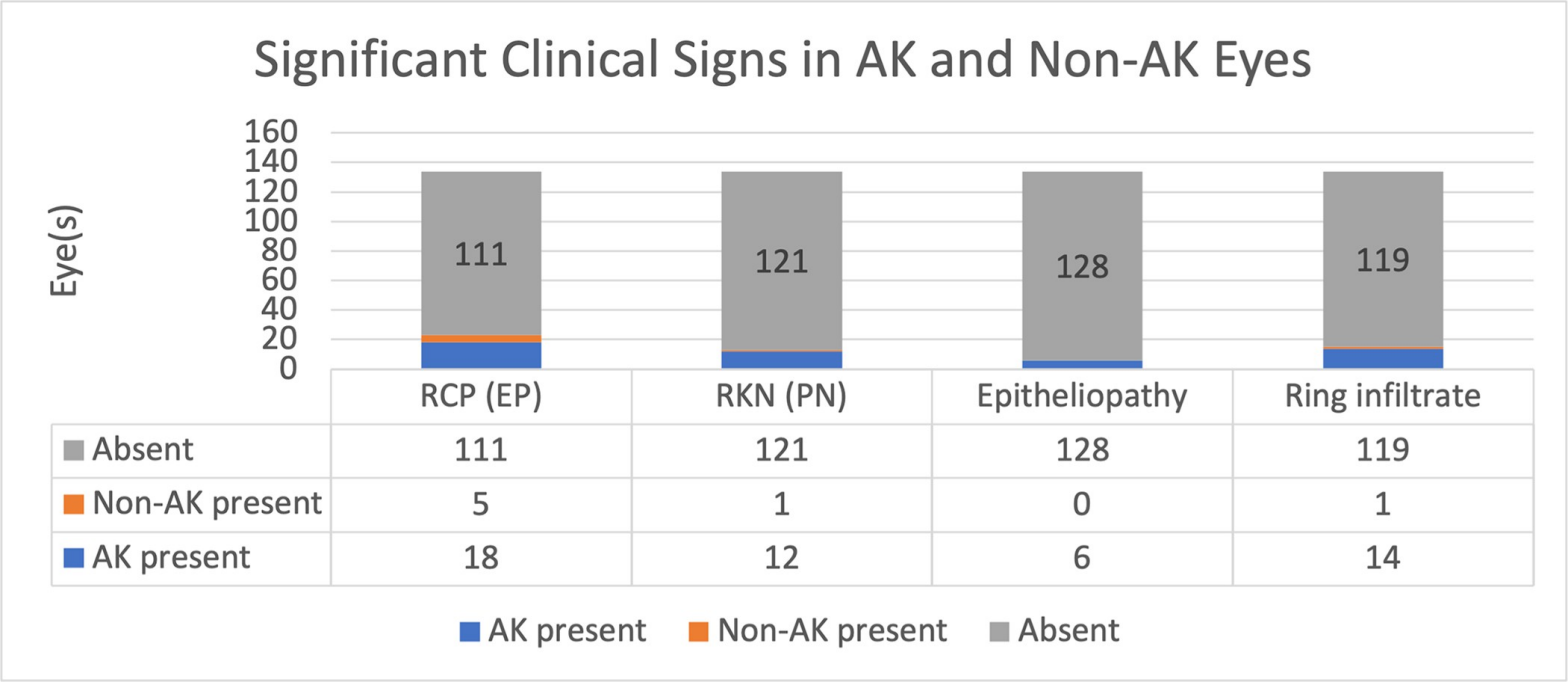
Comparison of significant clinical signs in acanthamoeba keratitis and non-acanthamoeba keratitis. RCP: Retro Corneal Plaques; EP: Endothelial Plaques; RKN: Radial Keratoneuritis; PN: Perineuritis; Non-AK: Non-Acanthamoeba Keratitis; AK: Acanthamoeba Keratitis.

More than half of the AK cases presented with ring infiltrate (53.8%), while (23.1%) had a stellate picture, and (23.1%) had an epitheliopathy picture. (Fig 3).

Fungal infection cases presented with combined dense infiltrate and stellate lesions in 41 eyes (95.3%). Herpetic cases presented with dendritic lesions in 38 eyes (88.3%). Bacterial cases were presented with dense infiltrate (87.3%).

The mean of the start time of radial keratoneuritis plaques ranged from day 0–30, with a mean (SD) of 3 ± 8.34 days; radial keratoneuritis was present for 7–40 days, with a mean (SD) of 12.85 ± 11.09 days, and resolved after a range of 7–45 days, with a mean (SD) of 27.85 ± 13.78 days. There is no significant difference between acanthamoeba keratitis and non-acanthamoeba keratitis cases. We also found that 76.9% of perineuritis (PN) was seen at first presentation, 91.9% in the 1st seven days, and one case on day 30. The mean duration of presence was 30.65 ± 11.09 days, ranging from 8–55 days, and the mean duration of plaques resolving was after 37.57 ± 11.58 days, a range of 8–60 days. There is no significant difference between acanthamoeba keratitis and non-acanthamoeba keratitis cases in terms of endothelial plaques occurrence time, duration, or resolving period. 21.7% of the endothelial plaques were found at the presentation. 65.2% were found in one week.

Based on culture, staining, and PCR results for patients with corneal infection and in those highly suspected of acanthamoeba infection, IVCM was performed. Acanthamoeba keratitis was confirmed only by staining in 6 cases (23%), confirmed only by culture in 6 cases (23%), and confirmed only by IVCM in 5 cases (19.2%). The bacterial cases were all confirmed with culture and staining. Most of the fungal cases were confirmed by culture (79%), while PCR confirmed 90.6% of herpetic cases.

## Discussion

In ophthalmology, infectious keratitis is a frequent emergency. The main culprits behind this condition are microorganisms, and it’s vital to determine which one is responsible in order to administer the most effective antimicrobial treatment and achieve the best possible results.

Infections of the cornea caused by Acanthamoeba can have various manifestations and require specific management strategies. Wide variations in the incidence of Acanthamoeba keratitis have been reported in both developed and developing countries, ranging from 1.4% to 2.5% [10–13]. The incidence increased over the years with the increase of contact lens wear, which was identified as a major risk factor [10, 14]. If left untreated, acanthamoeba can continue to grow deeper into the corneal stroma. This is because they feed on keratocytes, particularly in the deeper stroma, which can lead to corneal damage and resistance to treatment due to cyst formation and reduced drug penetration; thus, early treatment is crucial in preventing this from happening [2]. Misdiagnosing acanthamoeba is common as clinical signs and symptoms are difficult to distinguish from other organisms in early infection and can be associated with mixed organism infections in about one-third of the cases; a mixed infection with bacteria, and other organisms, including viruses or fungi, was reported [15, 16].

Studies have shown that the biggest risk factor for Acanthamoeba keratitis is contact lenses, with soft and daily disposable lenses being the most common culprits. This risk increases when contact lenses are misused or proper hygiene is not practiced, particularly when wearing them while bathing or cleaning them with tap water [17–22]. Our study showed that the most significant risk factor for acanthamoeba keratitis cases was the use of contact lenses. Furthermore, the majority of cases involving contact lens wearers were diagnosed earlier than those who did not wear contact lenses.

Radial keratoneuritis was reported as pathognomonic for Acanthamoeba keratitis; however, a similar appearance has also been reported in different etiologies of corneal infection, including bacteria and fungi, and other organisms [2, 23–26]. The incidence of radial keratoneuritis has been reported in 10% to 65.9% with Acanthamoeba keratitis [27, 28]. Nasef MH et al. reported that radial perineural corneal infiltrates were the most common ocular sign in patients with Acanthamoeba keratitis [28]. In addition, the incidence of radial keratoneuritis declines in cases diagnosed late to nearly half the percentage [29]. On the other hand, Alfawaz et al. reported that radial keratoneuritis can be the presenting sign [30]. We found that radial keratoneuritis is present in 13 eyes (9.7%) of all cases, 12 out of 13 cases (92.3%) of radial keratoneuritis were diagnosed as acanthamoeba keratitis, the only non-acanthamoeba case with radial keratoneuritis was diagnosed as herpetic keratitis confirmed with PCR and completely resolved with antiviral therapy; therefore, radial keratoneuritis is significantly associated with acanthamoeba keratitis (p<0.001).

Our findings align with previous studies, indicating that perineuritis (PN) was observed in 76.9% of cases at initial presentation. Additionally, 91.9% of cases were observed within the first seven days, with only one case reported on the 30^th^ day [28–30].

In our study, we found that 69.2% of AK cases are associated with retro-corneal plaques (RCP), and 78.3% of endothelial plaques are acanthamoeba keratitis cases (p<0.001), significantly associated with acanthamoeba keratitis. Endothelial plaques usually indicate a more severe infection and infiltration to the anterior chamber. They are usually observed as a typical finding of fungal keratitis [31–35]. Endothelial plaques are considered a sign of hyphae infiltrating Descemet’s membrane [36]. Acanthamoeba keratitis is known to infiltrate deep into the stroma; moreover, infiltration to the anterior chamber by acanthamoeba infection has been reported [37, 38].

**Fig 4:**
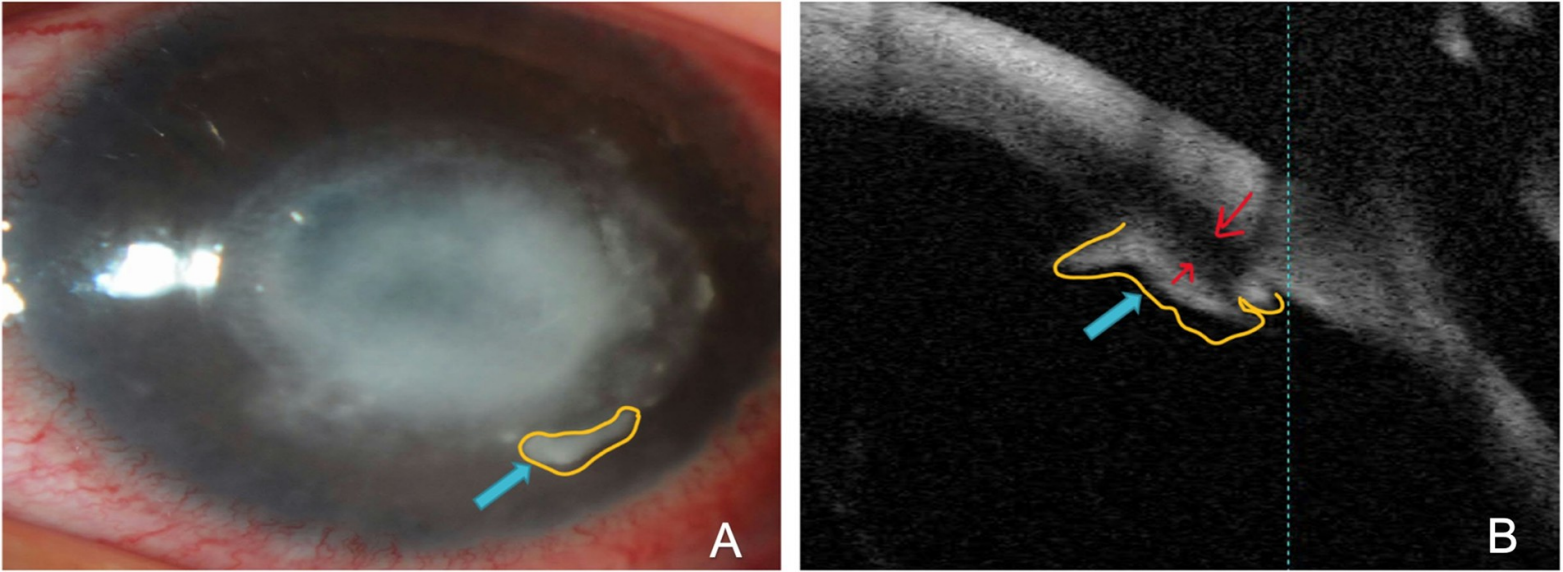
Slit-lamp photograph showing retro-corneal plaques (endothelial plaques) lesion associated with acanthamoeba keratitis showing more demarcated margins (yellow mark) (A); Retro-corneal plaques (Endothelial Plaques) in AS-OCT, fungal keratitis-associated endothelial plaques showing the absence of space (red arrows) and more demarcated margins (yellow mark) (B).

Takezawa et al. reported the anterior segment optical coherence tomography (AS-OCT) features that may help to differentiate the etiological organisms, mainly the presence of a clear boundary of the plaques and/or the presence of a space between the plaques and corneal endothelium supporting that is not extended from the corneal lesion rather than precipitation. Moreover, a space between the corneal endothelial surface and plaque was found in three patients with bacterial keratitis, and in fungal keratitis, an unclear boundary between the corneal endothelial surface and plaque, and high reflection of the plaque was extended from the corneal lesion [34]. This is consistent with the findings of Jin X et al. [39]. In our study, we noticed a similarity in AS-OCT findings of fungal keratitis-associated endothelial plaques regarding the absence of space (red arrows) and more demarcated margins (yellow mark). On the other hand, because of the insufficient number of AS-OCT tests in our data, we could not conclude and need further evaluation. (Fig 4)

Epitheliopathy in Acanthamoeba keratitis can be confused with herpetic epithelial keratitis. The absence of other associated signs makes the clinical diagnosis challenging [40, 41]. Acanthamoeba coinfection in the cornea has been reported in several studies with the presence of radial keratoneuritis and epitheliopathy [42–44]. We found only two Acanthamoeba coinfection cases, which were all with bacteria. Both were contact lens wearers and had endothelial plaques; one had radial keratoneuritis, and none had epitheliopathy. There was no difference in the presenting features, and it did not tend toward a worse clinical disease. Additionally, herpes simplex keratitis can be confused with early Acanthamoeba keratitis, and fungal keratitis can be confused with advanced stages of Acanthamoeba keratitis. In comparison with the herpetic and fungal infection, the presence of both radial keratoneuritis and endothelial plaques, in addition to the presence of epitheliopathy, is highly suggestive of acanthamoeba infection [15, 22, 45, 46].

Ring infiltrate, also known as Wessely immune ring, is from polymorphonuclear leukocytes, antigen-antibody-complex, and complement. It has been reported to develop in the first month in 20–50% of the patients, and the incidence increases with time. Although the Wessely immune ring may be present in bacterial, mycotic, or Acanthamoeba keratitis, the clinical image of the stromal infiltrates at the same time differentiates these clinical entities [2, 47–51]. The presence of ring infiltrate can be altered by the usage of topical steroids; because of its immune element, steroid reduces its incidence. The Wessely immune ring may develop within two days in patients with acanthamoeba keratitis after stopping topical steroids [48].

Overall, our sample had ring infiltrates in 15 out of the 134 eyes (11.2%). Of those 15 eyes, 14 eyes had acanthamoeba keratitis (93.3%), and one eye had herpetic confirmed with PCR. Of those, none of the cases with ring infiltrates were on topical steroids. All the ring infiltrates associated with acanthamoeba cases appeared at the time of presentation. Ring infiltrates were present on clinical exams for a mean of 37 days (ranging from 7–120 days). This is considered the most strongly associated sign of acanthamoeba keratitis in our study.

In addition to corneal scraping for staining and culture, IVCM is a highly effective tool for identifying Acanthamoeba [7]. IVCM is a highly sensitive, non-invasive imaging technique that is more effective for diagnosing acanthamoeba keratitis compared to PCR and culture methods [8, 9]. The challenge is the availability of the device and operator dependent. IVCM is both highly sensitive and specific when performed by an experienced specialist [4].

Another consideration is that Acanthamoeba cysts often form clusters after topical steroid use, and it is important to differentiate from the honeycomb distribution of the inflammatory cells in the anterior stroma in fungal keratitis [52]. IVCM is a useful tool in the evaluation of the management progress of acanthamoeba keratitis [53]. Table 1 demonstrates a comparison between different keratitis-causative organisms according to their relevant clinical signs.

**Table 1:** Comparison between different keratitis causative organisms based on the clinical signs.

Organism	Contact Lens Use	VA at presentation	IOP at presentation	Ring Infiltrate	Endothelial Plaques	Radial Keratoneuritis	Diagnostic Method
**Acanthamoeba**	****	20/100-20/200	Normal	****	****	****	IVCM, Culture, Staining,
**Bacterial**	*	20/100-20/200	Normal	-	**	None	Culture and staining
**Fungal**	*	<20/200	High IOP	-	*	None	Culture and staining
**Herpetic**	-	20/100-20/200	Normal	*	None	*	PCR

VA: Visual Acuity; IOP: Intraocular Pressure; IVCM: In-vivo Confocal Microscopy; PCR: Polymerase Chain Reaction.

*Represents the percentages of the associations that were concluded.

Fortunately, our study did not include a high number of keratitis that used steroids, with only 10 cases overall (7.5%). Out of the total cases of acanthamoeba keratitis, four cases (15% of AK) had been on topical steroids until the diagnosis was confirmed. On the other hand, this is an insufficient number and requires further investigation regarding the effect of steroids on keratitis prognosis. Most studies concluded that steroid use is probably best avoided [54]. Recent studies have reported an association between topical corticosteroids and delay of acanthamoeba keratitis diagnosis, which may worsen the prognosis [55, 56].

Our study limitations could include observer bias at the time of filling the medical records; this would be reduced by taking clinical photographs at the time of presentation as a more objective way of obtaining medical records data. Also, we had an insufficient number of cases that had AS-OCT testing and the number of patients who used steroid therapy.

## Conclusions

In this study, we concluded that the risk factors and the clinical features of acanthamoeba may aid in the early differentiation of acanthamoeba keratitis from other non-acanthamoeba keratitis. A higher suspicion of acanthamoeba keratitis was found in younger female patients and contact lens wearers. The presence of epitheliopathy, ring infiltrate, radial keratoneuritis, and endothelial plaques is highly suggestive of acanthamoeba infection, especially if combined signs are present. Herpes simplex keratitis can be confused with early Acanthamoeba keratitis, and fungal keratitis can be confused with the later stages of Acanthamoeba keratitis. Radial keratoneuritis and endothelial plaques may be useful clinical signs in evaluating the therapy progress. Although corneal scraping for culture and staining is the gold standard to diagnose infectious keratitis, the use of IVCM in suspected acanthamoeba infection or co-infection is important. The importance of promoting contact lens wear education is necessary as it is the most important risk factor of acanthamoeba infection to reduce the risk of infection.

## Supporting information

S1 Dataset The complete dataset used in this study. (XLSX)
